# Bioactive Metabolites from *Lactobacillus acidophilus*-Fermented Products Mitigate Carbon Tetrachloride–Induced Liver Injury: Biochemical and In Silico Insights

**DOI:** 10.1016/j.cdnut.2026.107648

**Published:** 2026-01-24

**Authors:** Md Shariful Islam, Md Monirul Islam, Abu Amer Hisham, SM Tanjil Shah, Md Arafat Al Mamun, Mohammad Salim Hossain

**Affiliations:** 1 Department of Pharmacy, Faculty of Biological Sciences, Noakhali Science and Technology University, Noakhali, Bangladesh; 2 Department of Microbiology, University of Dhaka, Dhaka, Bangladesh; 3 Fermentation and Pilot Plant Research Laboratory, Centre for Advanced Research in Sciences, University of Dhaka, Dhaka, Bangladesh

**Keywords:** functional foods, probiotic byproducts, dairy fermentation, nutritional bioactives, inflammatory mediators

## Abstract

**Background:**

Fermented products derived from *Lactobacillus* have demonstrated therapeutic potential in the management of metabolic disorders, particularly liver diseases.

**Objectives:**

This study aimed to evaluate the hepatoprotective effects of *Lactobacillus*-fermented products in a carbon tetrachloride (CCl_4_)-induced murine model of liver damage, with a focus on analyzing the influence of bioactive compounds on key liver function biomarkers.

**Methods:**

Two *Lactobacillus* strains (*Lactobacillus acidophilus* LB-CARS1 and *L. acidophilus* ST-CARS2) were isolated from commercial yogurts, and milk was fermented using each strain. A total of 40 male Swiss Albino mice were allocated into 5 experimental groups: control, CCl_4_-only, CCl_4_ + silymarin, and 2 treatment groups receiving CCl_4_ plus 1 of the 2 lactic acid bacteria (LAB)-fermented products. After 6 wk, serum concentrations of aspartate aminotransferase (AST), alanine transaminase (ALT), alkaline phosphatase (ALP), bilirubin, and creatinine were measured. A protein-protein network was constructed using the proteins responsible for hepatoprotection. The cellular pathways of these proteins were also visualized by the Kyoto Encyclopedia of Genes and Genomes (KEGG) pathway. Molecular docking was performed to assess interactions between identified bioactive compounds and target proteins (superoxide dismutase, catalase, transforming growth factor-β 1, peroxisome proliferator-activated receptor γ, IL-6, and tumor necrosis factor-*α*).

**Results:**

Treatment with LAB-fermented products significantly reduced (*P* < 0.05) serum AST, ALT, ALP, bilirubin, and creatinine concentrations in the CCl_4_-treated mice. The 2 products reduced the liver enzymes, though their intensity was different. The interactive pathways of the protein network revealed the most common genes responsible for hepatoprotective activity. Molecular docking revealed strong binding affinities between bioactive compounds and proteins related to inflammation and oxidation. Furthermore, absorption, distribution, metabolism, excretion, and toxicity analysis suggested the potential drug candidates among the docked compounds.

**Conclusions:**

The results indicate that LAB-fermented products contain bioactive compounds capable of attenuating liver injury, likely through anti-inflammatory and antioxidant mechanisms, highlighting their potential as functional food interventions in hepatoprotection.

## Introduction

The gut microbiota, a complex community of microorganisms residing in the gastrointestinal tract, is integral to host homeostasis, influencing metabolism, immunity, and barrier function [1,2]. Disruptions to this microbial equilibrium have been implicated in the pathogenesis of various diseases, including those affecting extraintestinal organs such as the liver. This relationship is mediated by the gut-liver axis, a bidirectional communication network through which gut-derived products, including microbial metabolites, can directly influence hepatic health [3,4].

Among the beneficial constituents of the gut microbiota are lactic acid bacteria (LAB), a group of gram-positive, nonspore-forming microorganisms. LAB, commonly found in fermented dairy, meat, and vegetable products, is recognized for its probiotic potential [5,6]. Notably, *Lactobacilli* can survive gastric transit to colonize the intestines, where they contribute to host health through competitive exclusion of pathogens, reinforcement of the epithelial barrier, and modulation of the immune system [7]. Specific strains, such as *Lactobacillus acidophilus*, *Lactobacillus rhamnosus*, and *Lactobacillus plantarum*, have demonstrated beneficial effects in mitigating metabolic disorders, including nonalcoholic fatty liver disease, often by restoring microbial balance and improving liver enzyme profiles [[8], [9], [10]]. Our previous research corroborates this, having improved liver enzyme profiles by feeding 2 LAB-fermented products in a model of high-fat diet-induced obesity [11].

The liver, as a central metabolic and detoxifying organ, is highly susceptible to injury from toxic substances [12]. Carbon tetrachloride (CCl_4_) is a well-characterized hepatotoxin used extensively to model chemical-induced liver injury [13]. Its mechanism of toxicity is 2-fold. First, cytochrome P450 (CYP450)-mediated metabolism of CCl_4_ generates highly reactive free radicals, which initiate lipid peroxidation, leading to oxidative stress, hepatocellular membrane damage, and ultimately, cell necrosis [14]. This oxidative insult depletes endogenous antioxidant defenses, such as superoxide dismutase (SOD). Second, the ensuing necroinflammation promotes the release of proinflammatory cytokines, including TNF-α, IL-6, and upregulates enzymes like cyclooxygenase-2. This cascade activates hepatic stellate cells, driving inflammation and fibrogenesis [15,16].

Although the beneficial effects of LAB on general liver health are acknowledged, their specific role and mechanisms in mitigating hepatotoxicity, particularly through their fermented products, remain insufficiently explored. The precise bioactive compounds responsible for these protective effects are largely undiscovered. Therefore, building on our previous findings, this study aimed to investigate the therapeutic potential of milk fermented with 2 locally isolated *Lactobacillus* strains against CCl_4_-induced acute hepatic injury in mice. We analyzed the chemical composition of the fermented products and evaluated their capacity to ameliorate liver damage by modulating oxidative stress and inflammatory responses.

## Methods

### Fermentation technique

Locally isolated LAB strains were identified as *L. acidophilus* via 16S rRNA sequencing and designated as *L. acidophilus* LB-CARS1 and ST-CARS2 (accession number: PV257720). Each strain was cultured individually on De Man-Rogosa–Sharpe agar for 24 h at 37°C. Subsequently, 2 distinct colonies of each strain were inoculated into separate containers of sterilized milk and fermented for 8 h at 37°C. The resulting fermented milk products were stored at −20°C until use. The compounds within the fermented products were characterized and reported previously [11].

### Ethical approval

The study was approved by the ethical review committee of Noakhali Science and Technology University (NSTU/SCI/EC/2023/143).

### Experimental animals

A total of 40 mature male Swiss Albino mice (6 wk old) were obtained from the Animal Resources Facility at the International Centre for Diarrheal Disease Research, Bangladesh (icddr,b). The mice were housed under controlled conditions (22 ± 1°C, 12-h light/dark cycle) with ad libitum access to food (icddr,b formulated rodent food pellet) and water. The pellet is composed of 53.85% carbs, 19.7% protein, 15.75% fat, 4.2% fiber, and 6.5% ash [17]. Bedding was changed regularly to maintain hygiene. After a 7-d acclimatization period, the mice were randomly (number generator) allocated into 5 experimental groups (*n* = 8) for a 5-wk intervention:

CD: control diet.

CCl_4_: administered a CD and received carbon tetrachloride (1 ml/kg of a 1:1 v/v mixture in olive oil, orally every third day).

CCl_4_ + SILM: received CCl_4_ (as above) plus silymarin (50 mg/kg, oral gavage, daily).

CCl_4_ + CARS1: received CCl_4_ (as above) plus the *L. acidophilus* LB-CARS1 fermented product (0.5 ml, oral gavage, daily).

CCl_4_ + CARS2: received CCl_4_ (as above) plus the *L. acidophilus* ST-CARS2 fermented product (0.5 ml, oral gavage, daily).

The principal investigator was aware of the group allocation during the different stages of the study.

### Biochemical analysis

On completion of the experimental protocol, all the mice were given a control diet (CD) for 48 h. After that, the mice were euthanized, and serum was collected. Hepatic enzyme profiles, including aspartate aminotransferase (AST), alanine transaminase (ALT), and alkaline phosphatase (ALP), were quantified in serum using commercial assay kits (Human Diagnostics) according to the manufacturer’s instructions. Furthermore, bilirubin and creatinine concentrations were also measured. Absorbance was measured using a semi-automatic Thermo Scientific Multiskan FC Microplate Photometer.

Data are expressed as the mean ± SEM. Statistical significance was determined by one-way analysis of variance followed by Tukey’s honest significant difference post hoc test, implemented in GraphPad Prism software (v8.00). Differences were considered significant at ∗*P* < 0.05, ∗∗*P* < 0.01, and ∗∗∗*P* < 0.001.

### Network pharmacology

A comprehensive list of 278 genes associated with hepatoprotection was generated from the GeneCards database using the keyword “hepatoprotective” [18]. The list was refined to the top 50 genes based on the GeneCards Inferred Functionality Score to prioritize the most functionally relevant candidates. These 50 genes were used as input for the STRING database to build a protein-protein interaction network and identify potential functional partnerships [19]. The resulting network was analyzed to discern significantly enriched pathways and the central genes within them. Reactome pathway analysis was further used to characterize the functional relationships between the involved biological processes [20].

### Molecular docking

The proteins responsible for hepatoprotective activity were docked against the compounds found in the fermented products. Catalase and SOD are antioxidant enzymes that protect the liver by neutralizing free radicals [21]. Transforming growth factor-β 1 (TGF-β1) plays a crucial role in liver repair and regeneration [22,23]. The peroxisome proliferator-activated receptor γ (PPAR-γ) generally exerts anti-inflammatory effects by suppressing the TNF-α and IL-6 in liver diseases [[24], [25], [26]]. These proteins were also found in the network pharmacology step. The standard ligands of inhibitors of the respective proteins were selected from the literature review. Protein structures were obtained from the protein data bank (PDB) and processed in PyMOL 2.5.7.0 to remove heteroatoms, ligands, and water molecules before saving in PDB format [27]. Energy minimization was performed using Swiss-PdbViewer 4.10. The compounds present in the fermented milk were identified using GC-MS, as reported in our previous study [11]. The refined proteins were docked with the compounds using PyRx, with ligand structures retrieved as 3D structure-data file (SDF) from PubChem [28,29]. Binding energies were recorded, and the best-scoring poses were visualized using Biovia Discovery Studio to analyze molecular interactions in 2-dimensional and 3-dimensional views.

### Absorption, distribution, metabolism, excretion, and toxicity analysis

Evaluation of pharmacokinetic parameters is essential for determining the therapeutic potential of chemical compounds. In silico absorption, distribution, metabolism, excretion, and toxicity (ADMET) analysis provides an efficient approach to predict these pharmacokinetic profiles. The ADMET study of the selected compounds was performed using the ADMETlab server [30]. This platform predicts key parameters, including human intestinal absorption (HIA), Caco-2 cell permeability, (CYP450) metabolism, volume of distribution (*V*_*D*_), blood-brain barrier (BBB) permeability, clearance, and various toxicity endpoints. The simplified molecular input line entry system (SMILES) representations of the compounds were submitted to the server, and the predicted pharmacokinetic and toxicity data were extracted for further interpretation.

## Results

### Effect on hepatic enzymes

**FIGURE 1:**
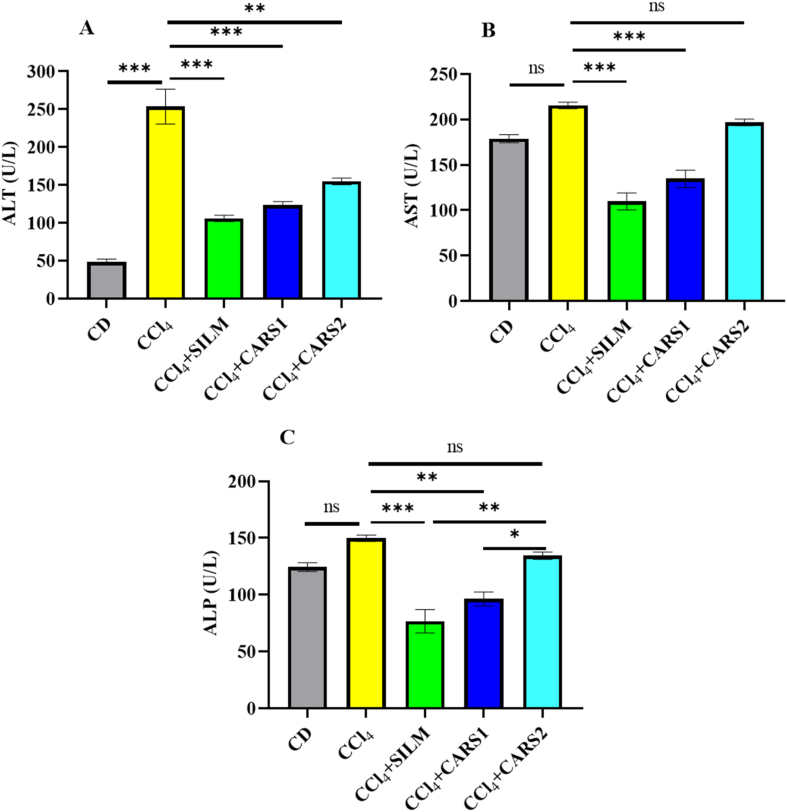
Serum concentrations of liver enzymes in different groups: (A) ALT, (B) AST, and (C) ALP. Data presented as mean ± SEM (*n* = 8). Significantly different at ∗*P* < 0.05, ∗∗*P* < 0.01, ∗∗∗*P* < 0.001. ALP, alkaline phosphatase; ALT, alanine transaminase; AST, aspartate aminotransferase; CD, control diet; CCl_4_, carbon tetrachloride; CCl_4_ + SILM, CCl_4_ + silymarin; CCl_4_ + CARS1, CCl_4_ + *Lactobacillus acidophilus* LB-CARS1 fermented product; CCl_4_ + CARS2, CCl_4_ + *L. acidophilus* ST-CARS2 fermented product; ns, nonsignificant.

Hepatic enzyme concentrations are established biomarkers of liver injury [31]. As anticipated, administration of CCl_4_ induced a pronounced elevation in serum concentrations of ALT, AST, and ALP in the model group. Treatment with *L. acidophilus* LB-CARS1 fermented milk significantly (*P* < 0.01) attenuated this increase, markedly reducing all 3 enzyme concentrations compared with the CCl_4_-intoxicated group (Figure 1). In contrast, treatment with *L. acidophilus* ST-CARS2 fermented milk produced a significant reduction (*P* < 0.01) only in ALT concentrations (Figure 1A). Furthermore, a statistically significant (*P* < 0.05) difference in ALP activity was observed between the 2 treatment groups themselves (Figure 1C).

### Effect on the bilirubin and creatinine concentrations

**FIGURE 2:**
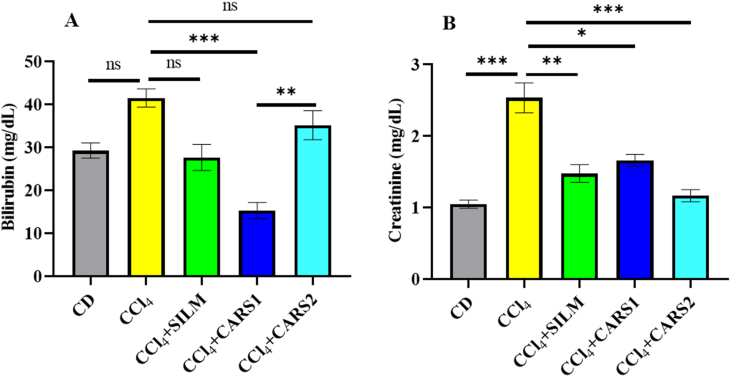
Serum concentrations of (A) bilirubin and (B) creatinine in different groups. Data presented as mean ± SEM (*n* = 8). Significantly different at ∗*P* < 0.05, ∗∗*P* < 0.01, ∗∗∗*P* < 0.001. CD, control diet; CCl_4_, carbon tetrachloride; CCl_4_ + SILM, CCl_4_ + silymarin; CCl_4_ + CARS1, CCl_4_ + *L. acidophilus* LB-CARS1 fermented product; CCl_4_ + CARS2, CCl_4_ + *L. acidophilus* ST-CARS2 fermented product; ns, nonsignificant.

The bilirubin and creatinine concentrations were increased in the CCl_4_-administered mouse group (Figure 2). The *L. acidophilus* LB-CARS1 showed a significant (*P* < 0.001) decrease in the bilirubin concentration, which was lower than that of the silymarin group. However, *L. acidophilus* ST-CARS2 significantly (*P* < 0.001) decreased the creatinine concentration, even lower than that of the silymarin group.

### Protein-protein network

**FIGURE 3:**
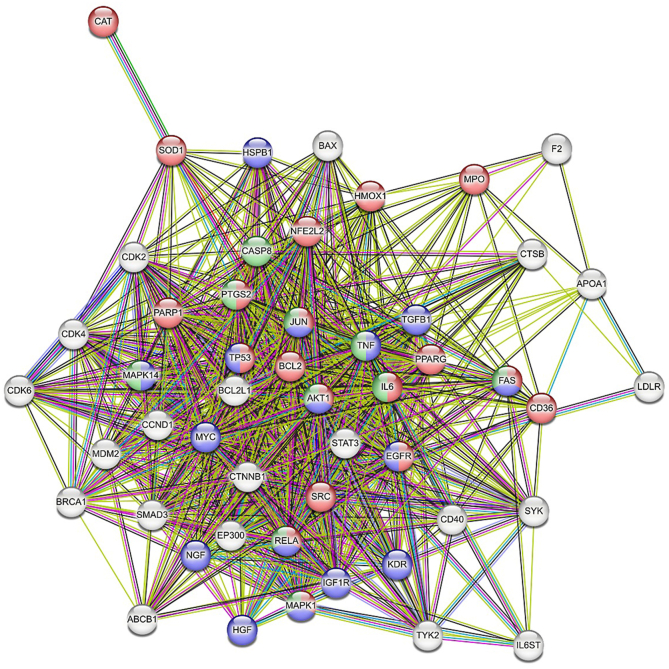
Network of genes responsible for hepatoprotection. Number of nodes = 50, number of edges = 724, mean node degree = 29, and mean local clustering coefficient = 0.812.

The protein-protein network constructed using the top genes is displayed in Figure 3. The network has significantly more interactions among the proteins than a random set from the genome.

**FIGURE 4:**
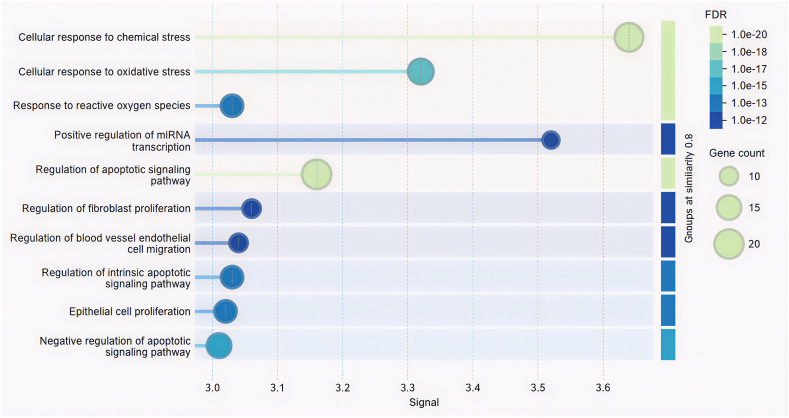
Top biological processes involved in the hepatoprotective activity. FDR, false discovery rate; miRNA, micro RNA.

Gene ontology analysis, performed after functional enrichment, revealed the predominant biological processes implicated in the network. As illustrated in Figure 4, these processes represent core cellular functions that modulate the central pathways responsible for liver protection.

Cellular response to chemical stress was the most prominently enriched biological process, based on both the significance of the signal and the number of implicated genes. The genes responsible for the top 5 processes are given in Table 1.

**TABLE 1:** Genes involved in the biological processes of hepatoprotection.

Biological processes	Genes involved
Cellular response to chemical stress	AKT1, BCL2, CAT, CD36, EGFR, FAS, HMOX1, IL6, JUN, MAPK1, MPO, NFE2L2, PARP1, PPARG, PTGS2, RELA, SOD1, SRC, TP53
Cellular response to oxidative stress	AKT1, BCL2, CAT, CD36, EGFR, HMOX1, IL6, JUN, MAPK1, MPO, NFE2L2, PARP1, RELA, SOD1, SRC, TP53
Response to reactive oxygen species	AKT1, BCL2, CAT, EGFR, HMOX1, IL6, JUN, MAPK1, MPO, NFE2L2, RELA, SOD1, SRC
Positive regulation of miRNA transcription	JUN, MYC, PPARG, RELA, SMAD3, STAT3, TGFB1, TNF, TP53
Regulation of the apoptotic signaling pathway	AKT1, BAX, BCL2, BCL2L1, BRCA1, CTNNB1, FAS, HGF, HMOX1, HSPB1, MDM2, MYC, NFE2L2, PARP1, PTGS2, RELA, SOD1, SRC, TNF, TP53

Abbreviations:AKT1, AKT serine/threonine kinase 1; AP-1, activator protein-1 transcription factor; BAX, BCL2-associated X apoptosis regulator; BCL2, B-cell CLL/lymphoma 2; BCL2L1, BCL2-like 1; BRCA1, breast cancer gene 1; CAT, catalase; CD36, cluster of differentiation 36; CTNNB1, catenin β 1; EGFR, epidermal growth factor receptor; FAS, Fas cell surface death receptor; HGF, hepatocyte growth factor; HMOX1, heme oxygenase 1; HSPB1, heat shock protein family B (small) member 1; JUN, Jun proto-oncogene; MAPK1, mitogen-activated protein kinase 1; MDM2, MDM2 proto-oncogene; miRNA, microRNA; MPO, myeloperoxidase; MYC, MYC proto-oncogene; NFE2L2, NFE2-like BZIP transcription factor 2; PARP1, poly(ADP-ribose) polymerase 1; PPARG, peroxisome proliferator-activated receptor γ; PTGS2, prostaglandin-endoperoxide synthase 2; RELA, RELA proto-oncogene, NF-κB subunit; SMAD3, SMAD family member 3; SOD1, superoxide dismutase 1; SRC, SRC proto-oncogene; STAT3, signal transducer and activator of transcription 3; TGFB1, transforming growth factor β 1; TP53, tumor protein p53.

**FIGURE 5:**
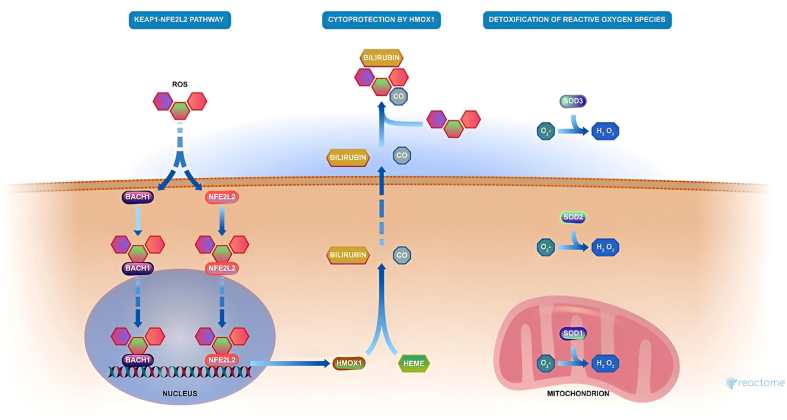
Pathways involved in the cellular response to the chemical stress. BACH1, BTB and CNC homology 1; HEME, iron-containing porphyrin ring structure; ROS, reactive oxygen species; SOD, superoxide dismutase.

The network between the genes of these subcellular processes is given in Supplementary Figures 1, 2, and 3. The pathway of response to chemical stress is displayed using Reactome (Figure 5). The other important pathways are displayed in the Supplementary Figures 4 and 5.

### Molecular docking

The top 10 compound-protein binding energies are summarized in Table 2. Among the tested compounds, 1-methylnaphthalene exhibited the most favorable binding energy with catalase, whereas 6-tert-butyl-3-(4,5-dihydro-1H-imidazol-2-ylmethyl)-2,4-dimethylphenol showed the strongest interaction with SOD. Similarly, 4-butan-2-yl-2,6-ditert-butylphenol demonstrated the lowest binding energy with TGF-β1, and 2-tert-butyl-5-[(3-methoxyphenyl) methylidene]-6-methyl-1,3-dioxan-4-one displayed the most favorable binding with PPAR-γ, TNF-α, and IL-6. Notably, all lowest binding energies were lower than those of the respective standard ligands, except in the case of TNF-α.

**TABLE 2:** Binding energies of the compounds with the studied proteins.

Compounds (PubChem ID)	Binding energy (kcal/mol)					
	Catalase	SOD	TGF-ß 1	PPAR-γ	TNF-α	IL-6
4 amino antipyrine (2151)	−8.3	—	—	—	—	—
Ascorbic acid (155903693)	—	−5.0	—	—	—	—
Galunisertib (10090485)	—	—	−7.8	—	—	—
Avicularin (5490064)	—	—	—	−8.6	—	—
SPD-304 (5327044)	—	—	—	—	−8.1	—
Resveratrol (445154)	—	—	—	—	—	−6.0
2-tert-butyl-5-[(3-methoxyphenyl) methylidene]-6-methyl-1,3-dioxan-4-one (5373757)	−8.3	−6.2	−7.7	−8.3	−7.9	−6.5
1-methylnaphthalene (7002)	−8.5	−5.5	−7.2	−6.7	−5.6	−5.8
1-(3,5-ditert-butyl-4-hydroxyphenyl) propan-1-one (616172)	−6.9	−6.0	−7.8	−7.3	−7.4	−5.2
6-tert-butyl-3-(4,5-dihydro-1H-imidazol-2-ylmethyl)-2,4-dimethylphenol (4636)	−6.8	−6.4	−7.4	−7.4	−7.6	−5.7
4-butan-2-yl-2,6-ditert-butylphenol (86583)	−6.0	−6.1	−8.1	−7.5	−7	−5.6
Naphthalene (931)	−7.8	−5.1	−6.9	−6.4	−5.8	−5.3
2,5-di*tert*-butylcyclohexa-2,5-diene-1,4-dione (17161)	−6.9	−5.4	−7.3	−6.9	−6.8	−6.0
2,6-ditert-butylphenol (31405)	−7.0	−5.5	−6.7	−6.9	−6.3	−5.5
benzoic acid (243)	−6.4	−4.8	−6.1	−5.5	−5.7	−5.3
2-phenylethanol (6054)	−6.2	−4.5	−6.0	−5.2	−5.3	−5.1

Abbreviations: PPAR-*γ*, peroxisome proliferator-activated receptor γ; SOD, superoxide dismutase; TGF-β1, transforming growth factor-β 1.

**FIGURE 6:**
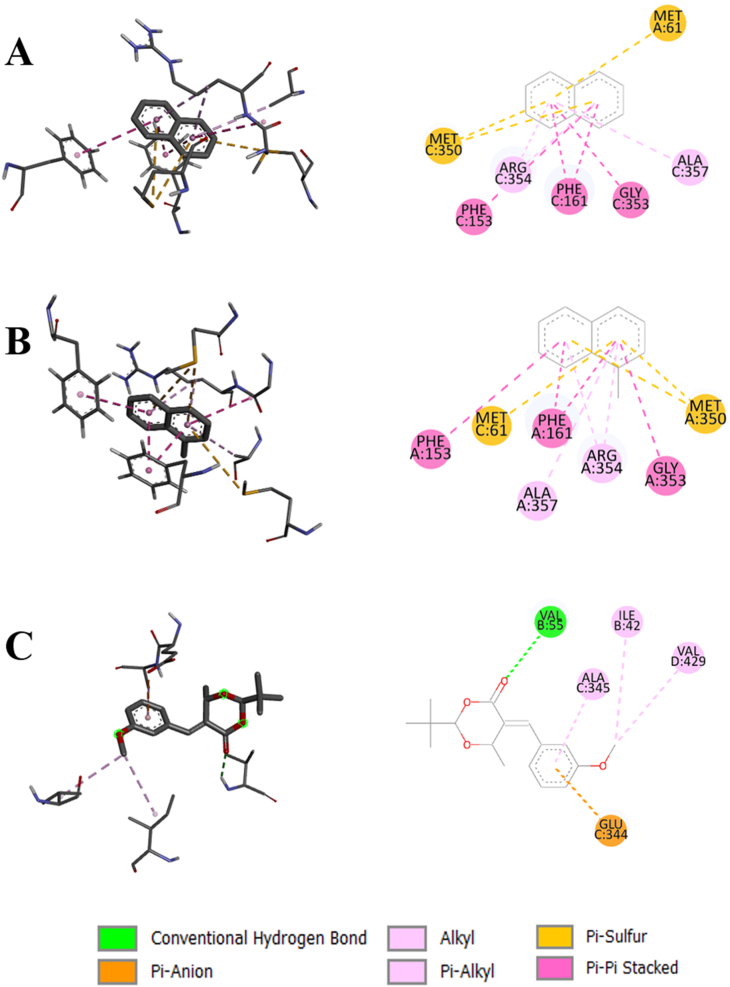
2D and 3D views of compounds interaction with catalase. (A) naphthalene (931), (B) 1-methylnaphthalene (7002), and (C) 2-tert-butyl-5-[(3-methoxyphenyl) methylidene]-6-methyl-1,3-dioxan-4-one (5373757).

The 2D and 3D views of the protein–ligand interactions are useful in depicting the types of interactions and the positions of bonds. Figure 6 shows the top 3 compound interactions with the catalase. The interactions with these compounds are mainly of conventional hydrogen and hydrophobic bonds.

**FIGURE 7:**
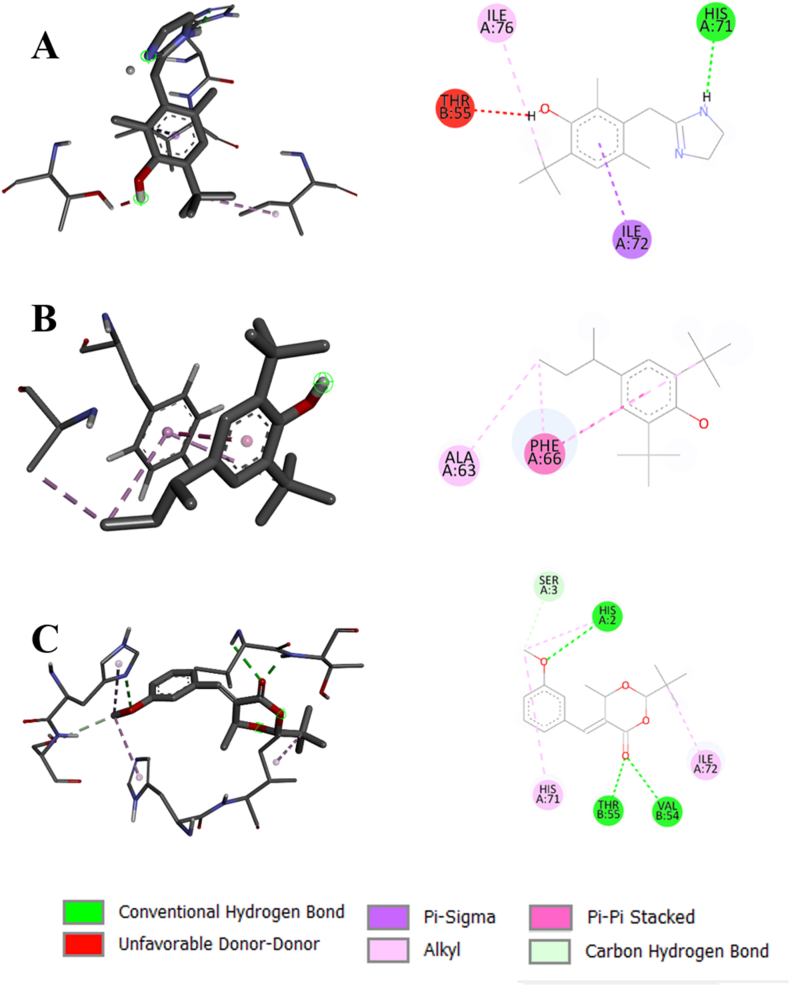
2D and 3D views of compounds interaction with SOD. (A) 6-tert-butyl-3-(4,5-dihydro-1H-imidazol-2-ylmethyl)-2,4-dimethylphenol (4636), (B) 4-butan-2-yl-2,6-ditert-butylphenol (86583), and (C) 2-tert-butyl-5-[(3-methoxyphenyl) methylidene]-6-methyl-1,3-dioxan-4-one (5373757). SOD, superoxide dismutase.

**FIGURE 8:**
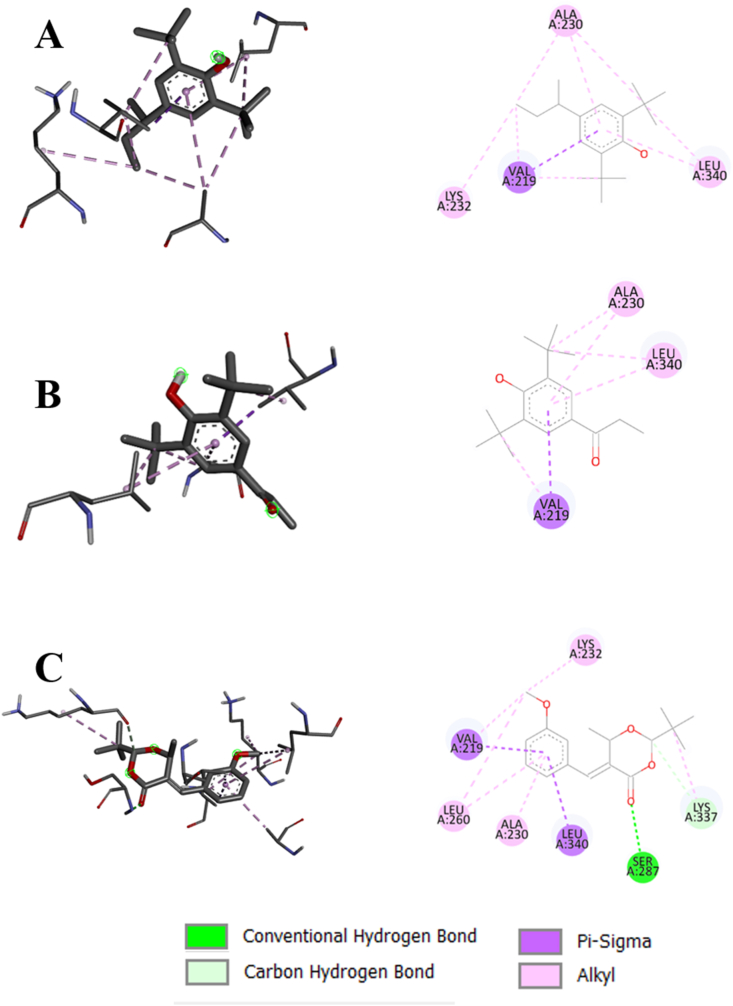
2D and 3D views of compounds interaction with TGF-β1, (A) 4-butan-2-yl-2,6-ditert-butylphenol (86583), (B) 1-(3,5-ditert-butyl-4-hydroxyphenyl) propan-1-one (616172), and (C) 2-tert-butyl-5-[(3-methoxyphenyl) methylidene]-6-methyl-1,3-dioxan-4-one (5373757). TGF-β1, transforming growth factor-β 1.

The standard ligand (4-amino antipyrine) binds with the catalase at the positions of ASN148, PHE153, PHE161, MET350, GLY353, and ARG354. Naphthalene also shows bonds with PHE153, PHE161, GLY353, and ARG354, along with some additional interactions with other amino acids. Similarly, 1-methylnaphthalene formed bonds with PHE153, PHE161, MET350, GLY353, and ARG354. This implies that these compounds have almost similar binding properties to the standard ligand with the target proteins. However, 2-tert-butyl-5-[(3-methoxyphenyl) methylidene]-6-methyl-1,3-dioxan-4-one formed bonds with GLU344 and ALA345, which are near the standard binding region. Figure 7 depicts the interaction of SOD with the top 3 compounds. 6-tert-butyl-3-(4,5-dihydro-1H-imidazol-2-ylmethyl)-2,4-dimethylphenol formed conventional hydrogen bond with HIS71, hydrophobic bonds with ILE72, ILE76, and unfavorable bond with THR55. On the other hand, the 4-butan-2-yl-2,6-ditert-butylphenol showed only hydrophobic bonds with ALA63 and PHE66. However, 2-tert-butyl-5-[(3-methoxyphenyl) methylidene]-6-methyl-1,3-dioxan-4-one exhibited conventional hydrogen bonds with HIS2, VAL54, THR55, HIS71, ILE72, carbon hydrogen bond with SER3, and hydrophobic bonds with HIS2, HIS71, ILE72. Figure 8 displays the interaction of TGF-ß 1 with the top 3 compounds. The standard ligand (Galunisertib) forms a hydrophobic bond at the VAL219 and LYS337 positions. 4-butan-2-yl-2,6-ditert-butylphenol and 1-(3,5-ditert-butyl-4-hydroxyphenyl) propan-1-one also form hydrophobic bond at the VAL219 position. However, 2-tert-butyl-5-[(3-methoxyphenyl) methylidene]-6-methyl-1,3-dioxan-4-one forms the bonds similar to the standard, along with some additional bonds. The binding interactions with the PPAR-*γ*, TNF-*α*, and IL-6 are given in Supplementary Figures 6–8.

### ADMET analysis

The result of the ADMET analysis is shown in Table 3. The important ADMET properties of the top binding compounds are included in the table. The ranges indicate the probability of being the properties of the compound. The optimum logP value ranges from 0 to 3, and logS ranges from –4 to 0.5. Based on these criteria, compounds having PubChem identifications 243 and 6054 are in the ranges. The Caco-2 permeability value shows that all the compounds are within a moderate value (>−5.15) except the compound 86583. Plasma protein binding percentage shows compounds 4636, 243, and 6054 have <90% plasma protein binding value. The *V*_*D*_ (0.04–20) and creatinine clearance (5–15) values were optimum for all compounds. The probability of oral toxicity and carcinogenicity was also low for most of the compounds. All the compounds fulfilled the Lipinski drug rules.

**TABLE 3:** ADMET properties of the bioactive compounds from *Lactobacillus*-fermented products.

CID	LogP	LogS	HIA (probability of HIA)	Caco-2	BBB (probability of being BBB+)	PPB (%)	*V*_*D*_ (L/kg)	CYP3A4 inhibitor	CYP3A4 substrate	C_L_ (mL/min/kg)	*T*_1/2_ (probability of being long half-life, >3 h)	Rat oral acute toxicity (probability of being toxic)	Carcinogenicity (probability of being carcinogenic)	Lipinski
5373757	3.806	−4.851	0–0.1	−4.591	0.1–0.3	94.24	1.382	0.1–0.3	0.5–0.7	6.668	0.155	0–0.1	0.1–0.3	Accepted
7002	3.802	−4.213	0–0.1	−4.345	0.5–0.7	95.83	1.174	0.1–0.3	0.1–0.3	9.868	0.306	0–0.1	0.7–0.9	Accepted
616172	4.845	−5.047	0.3–0.5	−5.134	0.3–0.5	98.56	1.114	0.3–0.5	0.5–0.7	3.902	0.418	0.1–0.3	0–0.1	Accepted
4636	3.823	−3.578	0–0.1	−5.062	0.1–0.3	65.13	2.393	0–0.1	0.5–0.7	14.81	0.826	0.7–0.9	0.1–0.3	Accepted
86583	6.21	−5.352	0.7–0.9	−5.322	0.3–0.5	100.32	5.571	0.5–0.7	0.7–0.9	4.07	0.117	0.1–0.3	0–0.1	Accepted
931	3.302	−3.657	0–0.1	−4.237	0.5–0.7	95.69	1.177	0–0.1	0.1–0.3	10.47	0.399	0–0.1	0.7–0.9	Accepted
17161	3.964	−4.936	0–0.1	−4.834	0–0.1	93.16	1.924	0.5–0.7	0.3–0.5	3.033	0.526	0.7–0.9	0.7–0.9	Accepted
31405	4.585	−4.714	0.5–0.7	−4.921	0.1–0.3	99.20	5.069	0.3–0.5	0.5–0.7	5.683	0.271	0.1–0.3	0–0.1	Accepted
243	1.957	−1.817	0–0.1	−4.942	0.3–0.5	58.28	0.208	0–0.1	0–0.1	3.574	0.909	0.1–0.3	0–0.1	Accepted
6054	1.372	−1.196	0–0.1	−4.168	0.9–1.0	53.13	4.266	0–0.1	0.3–0.5	10.085	0.826	0–0.1	0.3–0.5	Accepted

Abbreviations: ADMET, absorption, distribution, metabolism, excretion, and toxicity; BBB, blood-brain barrier; C_L_, clearance; CID, compound identification number; C_L_, clearance; CYP3A4, cytochrome P3A4; HIA, human intestinal absorption; PPB, plasma protein binding; *V*_*D*_, volume of distribution; *T*_1/2_, half-life.

## Discussion

*Lactobacillus* spp. ameliorate a spectrum of conditions, including gastrointestinal diseases, allergies, and liver disorders, through multiple mechanisms. These include the production of antimicrobial metabolites, direct immunomodulation, and competitive exclusion of pathogens to reshape the gut microbiota [32,33]. The gut-liver axis is a critical pathway through which gut microbes influence hepatic health, with probiotics demonstrating efficacy in both alcoholic and nonalcoholic liver disease. For instance, *Lactobacillus* intake counteracts high-fat diet-induced obesity, attenuates inflammation, restores a healthy gut microbiome, and enhances intestinal barrier function [34]. Notably, the beneficial effects of probiotics extend beyond viable bacteria to include their bioactive metabolites.

Serum ALT concentration is a sensitive biomarker for hepatocellular injury, as elevated concentrations reflect leakage of this enzyme from damaged hepatocytes into circulation [35]. As shown in Figure 2A, administration of CCl_4_ produced a marked elevation in ALT compared with the CD group (*P* < 0.001). Interestingly, both LAB-fermented products (CARS1 and CARS2) led to significant reductions in ALT concentrations relative to the CCl_4_ group (*P* < 0.001). Although the decrease was slightly less pronounced than with silymarin, the improvement was statistically meaningful, indicating that the bioactive metabolites derived from *L. acidophilus* fermentation exert protective effects against CCl_4_-induced hepatic damage. In case of AST, administration of CCl_4_ produced a notable, though statistically nonsignificant, increase in AST concentrations compared with the CD group (Figure 2B). This result suggests that although hepatotoxicity was induced, AST elevations may not have been as pronounced as ALT, possibly due to the extrahepatic distribution of AST and its lower specificity for liver injury [36]. The effect of silymarin and LAB-fermented products on the CCl_4_-induced toxicity was similar to that of ALT. As shown in Figure 2C, administration of CCl_4_ caused an observable increase in serum ALP compared with the CD group. Administration of CARS1-fermented products led to significant (*P* < 0.01) reductions in ALP concentrations. However, the other strain showed a reduction in the ALP amount compared with the CCl_4_ group, but not at a significant concentration. This observation aligns with previous reports that probiotic-fermented products can ameliorate liver dysfunction by modulating oxidative stress, inflammatory pathways, and the gut-liver axis. A growing body of evidence underscores the hepatoprotective efficacy of various *Lactobacillus* strains. For instance, *L. plantarum* HFY15 and *L. acidophilus* KLDS1.0901 were shown to ameliorate CCl_4_-induced liver injury in mice, as evidenced by a significant reduction in circulating hepatic enzyme concentrations [9,37]. Similarly, *L. acidophilus* LA14 attenuated hepatic damage in a d-galactosamine-induced rat model [38]. Proposed mechanisms for such protection, as demonstrated by *L. acidophilus*, include the modulation of bile acid homeostasis through the inhibition of synthesis and promotion of excretion [39]. Consistent with these findings, our study also revealed significant inter-strain differences in hepatoprotective activity based on attenuation of serum liver damage markers. Specifically, the strain *L. acidophilus* CARS1 conferred a more pronounced reduction in ALT and AST concentrations compared with others, suggesting that the observed liver enzyme reducing potential is highly strain-specific. This variation may be attributed to the differential production of bioactive metabolites during fermentation.

Bilirubin and creatinine are critical biochemical indicators for evaluating hepatic and renal function, respectively. Increased serum bilirubin concentrations reflect impaired hepatobiliary function, whereas elevated creatinine concentrations are associated with renal dysfunction [40,41]. In this study, administration of CCl_4_ resulted in a marked increase in both bilirubin and creatinine compared with the CD group. These findings are consistent with the well-documented hepatotoxic and nephrotoxic effects of CCl_4_, which involve the generation of free radicals and membrane lipid peroxidation [42]. Mice treated with LAB-fermented products (CARS1 and CARS2) also demonstrated a considerable decline in these biomarkers compared with the CCl_4_-only group. Among the 2, CARS1 showed the most pronounced reduction in bilirubin, whereas the CARS2 administration further reduced the creatinine concentration. These improvements can likely be attributed to the presence of bioactive metabolites generated during fermentation, which may act through antioxidant and anti-inflammatory mechanisms, stabilization of hepatocyte membranes, and modulation of gut-liver axis communication [43,44]. The observed effects align with previous reports that *Lactobacillus*-fermented foods enhance host antioxidant defenses, reduce lipid peroxidation, and improve detoxification pathways [45,46]. By lowering bilirubin and creatinine concentrations, LAB-fermented products demonstrated their capacity to act against CCl_4_-induced oxidative stress. In the absence of kidney-specific functional assays, the present results do not provide direct evidence of renal protection. Rather, the observed modulation of serum creatinine may reflect an indirect or secondary effect associated with overall attenuation of CCl_4_-induced toxicity. Further studies incorporating dedicated renal assessments are required to substantiate any potential nephroprotective mechanisms.

The in silico analyses performed in this study are inherently predictive and exploratory in nature. These computational approaches were used to generate hypotheses regarding potential molecular targets, signaling pathways, and bioactive compounds that may contribute to the observed hepatoprotective effects of the fermented products. Network pharmacology is a powerful tool for identifying key genes within biological processes. Our investigation into hepatoprotective mechanisms uncovered a substantial number of involved genes. Subsequent functional enrichment analysis highlighted the most highly interconnected gene set within this network. The most common genes involved in the network include AKT1, BCL2, CAT, EGFR, HMOX1, IL6, JUN, PPARG, SOD1, and SRC. The strong enrichment of processes like “Cellular response to oxidative stress” and “Response to reactive oxygen species” is highly significant. This strongly suggests that the mechanism of action for many of these hepatoprotective genes involves mitigating oxidative damage, a well-established pathway in liver injury caused by toxins. This enrichment analysis effectively moves from a simple gene list to a mechanistic model of hepatoprotection. The figure demonstrates that the identified genes are not random but are cohesively involved in a logical biological narrative: responding to chemical stress, combating oxidative damage, carefully regulating cell death, and promoting tissue repair and regeneration.

Molecular docking was performed to predict the interaction of bioactive compounds derived from LAB-fermented products with key targets associated with hepatoprotection, oxidative stress regulation, and inflammation. The binding energies obtained provide valuable insight into the potential mechanisms through which these compounds may exert hepatoprotective activity. Among the tested molecules, 2-tert-butyl-5-[(3-methoxyphenyl) methylidene]-6-methyl-1,3-dioxan-4-one (PubChem identification: 5373757) demonstrated high affinity across multiple targets, including catalase, SOD, TGF-β1, PPAR-γ, TNF-α, and IL-6. Similarly, 1-methylnaphthalene (PubChem identification: 7002) showed strong multitarget binding with favorable energies across the studied macromolecules. This broad-spectrum interaction suggests that it may play a key role in modulating oxidative stress enzymes while simultaneously attenuating proinflammatory signaling pathways. Several phenolic derivatives, including 1-(3,5-di-tert-butyl-4-hydroxyphenyl) propan-1-one, 6-tert-butyl-3-(imidazolylmethyl)-2,4-dimethylphenol, and 4-butan-2-yl-2,6-ditert-butylphenol, exhibited stable binding affinities in the range of −6.0 to −8.1 kcal/mol against multiple targets. These results suggest that *Lactobacillus* fermentation-derived phenolic metabolites may contribute significantly to the observed hepatoprotective effects by enhancing endogenous antioxidant defenses and mitigating proinflammatory signaling [47,48]. Taken together, these findings highlight that LAB-fermented products yield a diverse array of bioactive metabolites capable of modulating multiple targets that are associated with liver complications. The compounds not only interact with antioxidant enzymes (catalase, SOD) [49,50] but also strongly bind to inflammatory mediators (TNF-α, IL-6) [51,52] and regulators of fibrosis and lipid metabolism (TGF-β1, PPAR-γ) [23]. Such multitarget activity is highly desirable in hepatoprotection, given the multifactorial nature of liver injury involving oxidative stress, inflammation, and fibrosis.

The in silico ADMET profiling of the selected compounds provides crucial insights into their potential pharmacokinetic and toxicity profiles, enabling an early assessment of their drug-likeness and prioritization for further development. Overall, the compounds demonstrate favorable absorption and permeability characteristics. The majority exhibit a high probability of HIA, as indicated by low HIA probability scores for most compounds. This is further supported by the Caco-2 permeability values, which, although generally low (ranging from −4.2 to −5.3), are within an acceptable range for many orally administered drugs that rely on passive transcellular diffusion. All compounds comply with Lipinski’s Rule of Five, confirming their good oral bioavailability potential from a molecular weight and lipophilicity perspective [53]. The distribution properties, however, present a mixed profile. Most compounds show very high plasma protein binding, which could significantly reduce the free fraction of the drug available to exert a therapeutic effect and may necessitate higher dosing regimens [54]. The *V*_*D*_ values also varies widely. The potential to cross the BBB is generally low to moderate for most compounds, which is a desirable trait to avoid central nervous system (CNS)-related side effects for non-CNS targets [55]. A significant number of compounds are predicted to be substrates for the cytochrome P3A4 (CYP3A4) isoenzyme, indicating a potential for pharmacokinetic drug-drug interactions. Furthermore, some compounds show a probability of inhibiting CYP3A4, which raises an additional interaction risk that would require careful investigation in later stages. The total clearance values are largely low to moderate, and the half-life predictions suggest that most compounds have a short to medium half-life. Based on the toxicity profile, compound identification number 17161 may be a less favorable candidate due to its concurrent high-risk predictions for both acute toxicity and carcinogenicity. Conversely, several compounds (e.g., 616172, 86583, 31405, 243) present with low probabilities for both acute toxicity and carcinogenicity, marking them as safer candidates from this preliminary perspective. Although these findings provide a supportive mechanistic context and biological plausibility for the in vivo results, they do not constitute direct experimental validation of the proposed mechanisms. Further targeted molecular and functional studies will be required to confirm the involvement of the identified pathways and targets.

### Limitations

This study has several limitations. First, the hepatoprotective effects of the *L. acidophilus*-fermented products were evaluated at a single dose. Investigating a range of doses would be necessary to establish a dose-response relationship and determine the optimal therapeutic dosage. Second, due to constraints on resources and time, histopathological examination of liver tissue was not conducted; such analysis could have provided valuable morphological insights to corroborate the biochemical findings. The assessment of liver injury was primarily based on serum concentrations of ALT, AST, and ALP, without direct measurement of these enzymes in liver tissue homogenates. Moreover, mice exhibit significant sex-specific differences in liver diseases, but we used only male mice for our study. Finally, the in silico predictions of the mechanism were not validated through gene or protein expression analysis, which would strengthen the biological conclusions of the study. Future studies incorporating hepatic enzyme assays and molecular markers of fibrosis will be essential to further substantiate and mechanistically validate the findings reported here.

In conclusion, *L. acidophilus* is considered a useful probiotic for human health. The present study investigates the 2 local strains of *L. acidophilus*-fermented milk for their hepatoprotective effects in CCl_4_-injured mice. The fermented products substantially reduce the hepatic enzymes, bilirubin, and creatinine in the CCl_4_-administered mice. The protein-protein network revealed important protein targets for hepatoprotective activity. The molecular docking also revealed the strong binding affinity with hepatoprotective enzymes, antioxidants, and inflammatory cytokines. The ADMET analysis showed the potential lead compounds to be used as hepatoprotective agents. Overall, these results reinforce the hepatoprotective potential of LAB-fermented products and highlight their value as functional foods that may mitigate toxin-induced hepatic injury and its associated systemic complications. Future in vitro and in vivo validation studies are warranted to confirm these docking predictions and to identify the synergistic effects of these compounds in complex biological systems.

## Author contributions

The authors’ responsibilities were as follows – MSI, MMI, AAH: contributed in investigation, formal analysis, and writing—original draft; MMI: contributed in visualization and software; SMTS, MAAM: contributed in investigation, resources, and review and editing; MSH: contributed in conceptualization, methodology, writing—review and editing, and supervision; and all authors: read and approved the final manuscript.

## Data availability statement

Data described in the manuscript, code book, and analytic code will be made available upon request pending approval.

## Funding

This work was funded by the Research Cell of Noakhali Science and Technology University (NSTU/RC-PH-09/T-23/132).

## Conflict of interest

The authors report no conflicts of interest.
