# Supplementary material for: Bioactive Metabolites from Lactobacillus acidophilus-Fermented Products Mitigate Carbon Tetrachloride–Induced Liver Injury: Biochemical and In Silico Insights
Source: Curr Dev Nutr. 2026 Jan 24;10(2):107648. doi: 10.1016/j.cdnut.2026.107648 (PMC12925109; doi:10.1016/j.cdnut.2026.107648)
Supplement: multimedia component 1 [file mmc1.docx]

Md. Shariful Islam et al., Bioactive Metabolites from *Lactobacillus acidophilus*-Fermented Products Mitigate CCl_4_-Induced Liver Injury: Biochemical and *In-silico* Insights


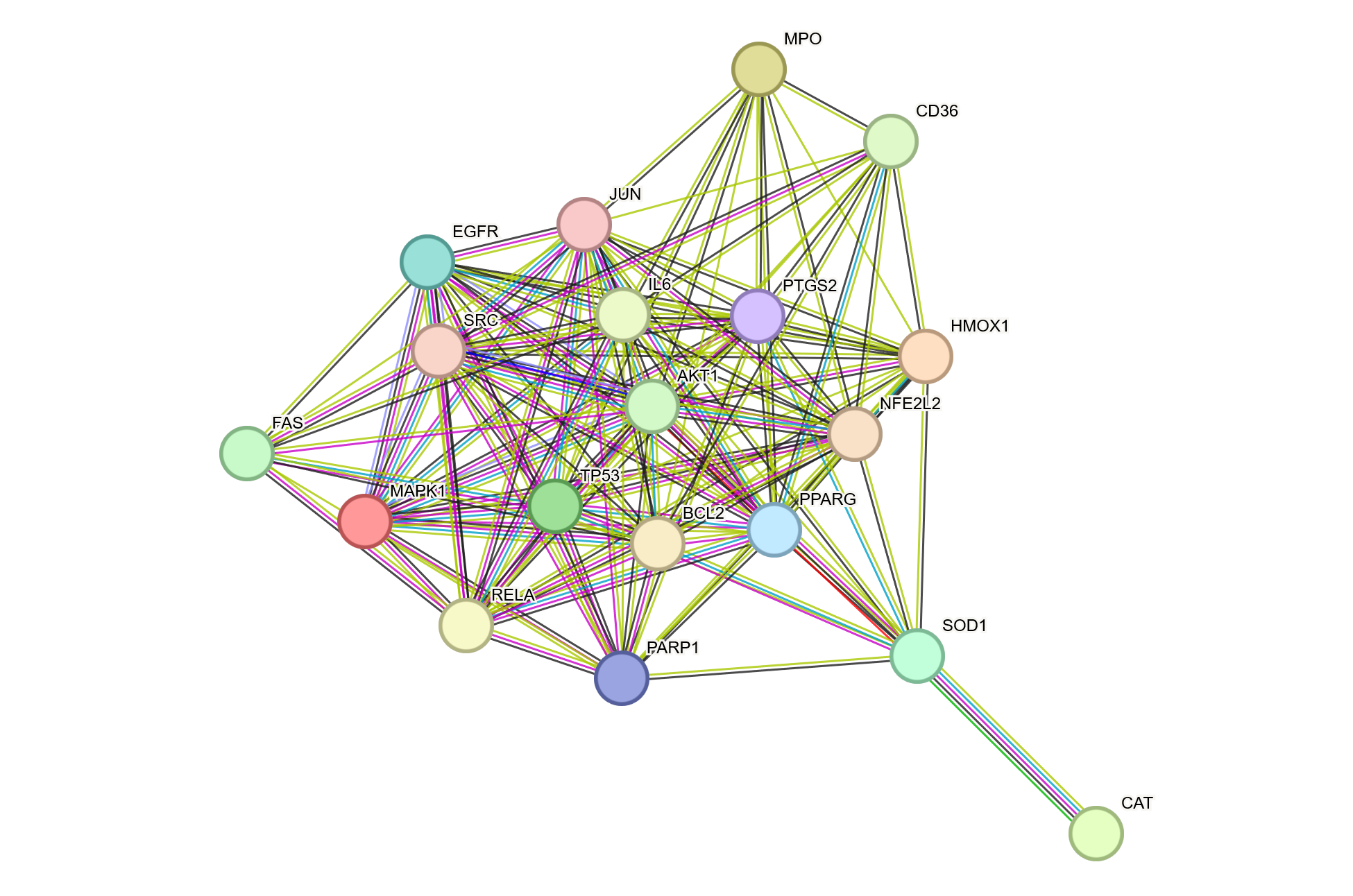


SF 1: Network among the genes involved in the cellular response to chemical stress


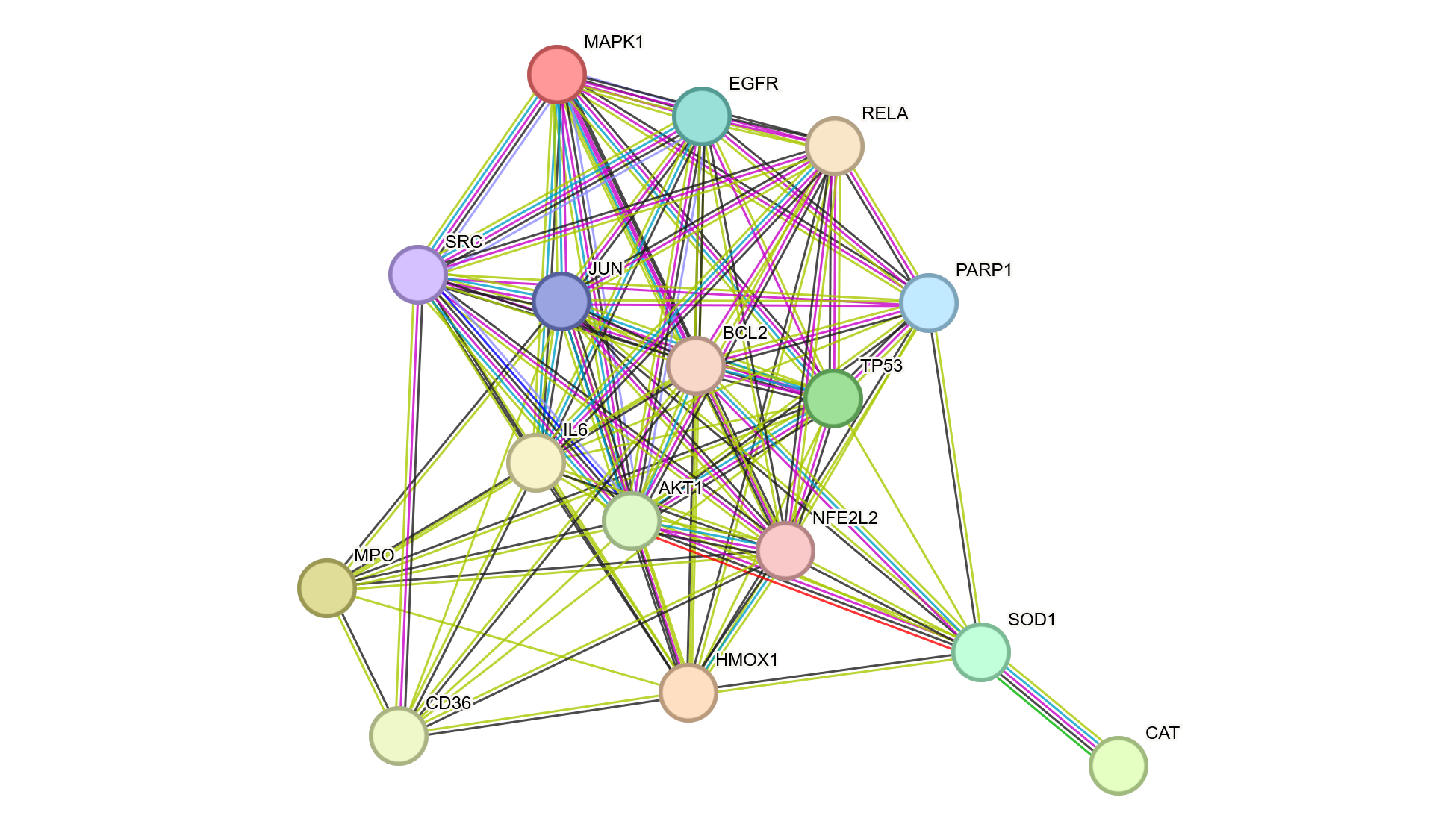


SF 2: Network among the genes involved in the cellular response to oxidative stress


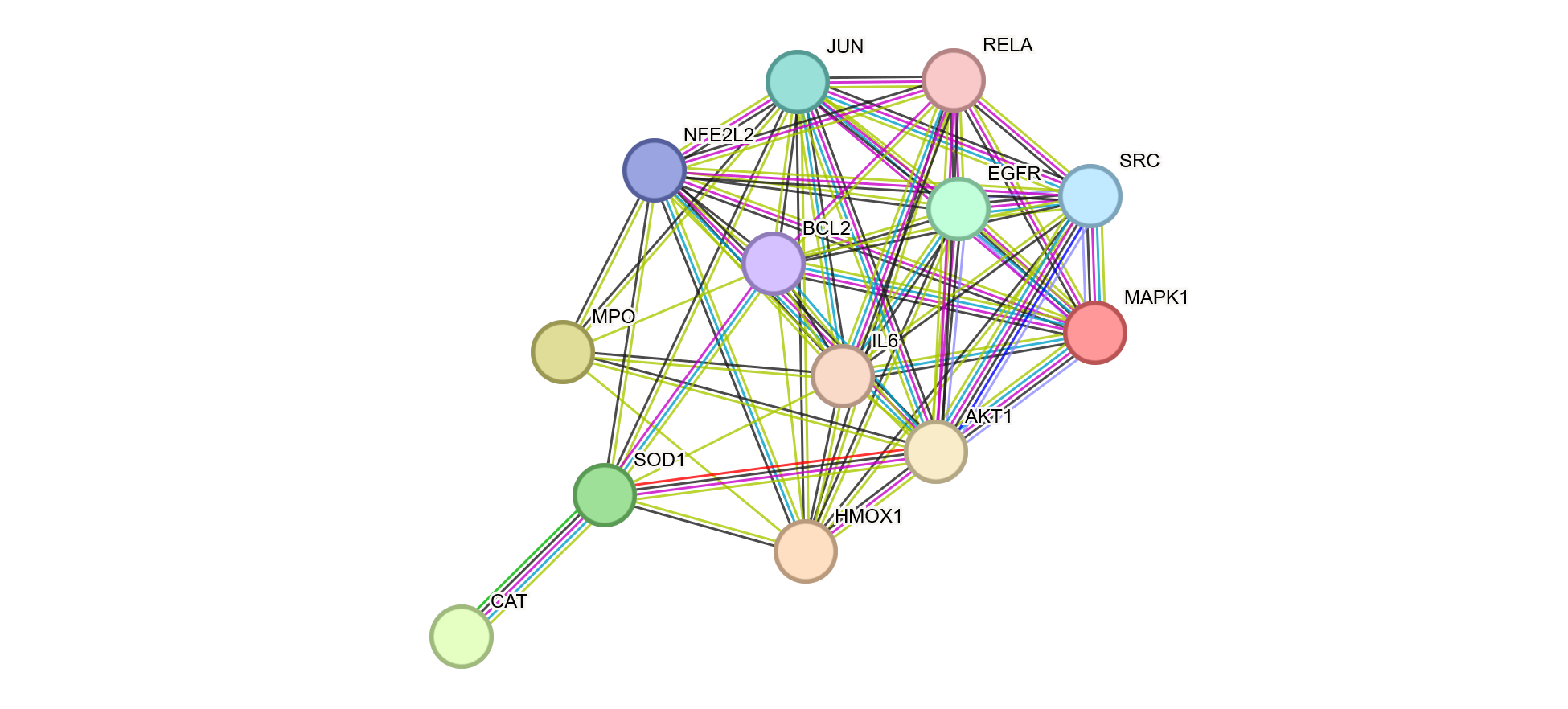


SF 3: Network among the genes involved in the cellular response to reactive oxygen species


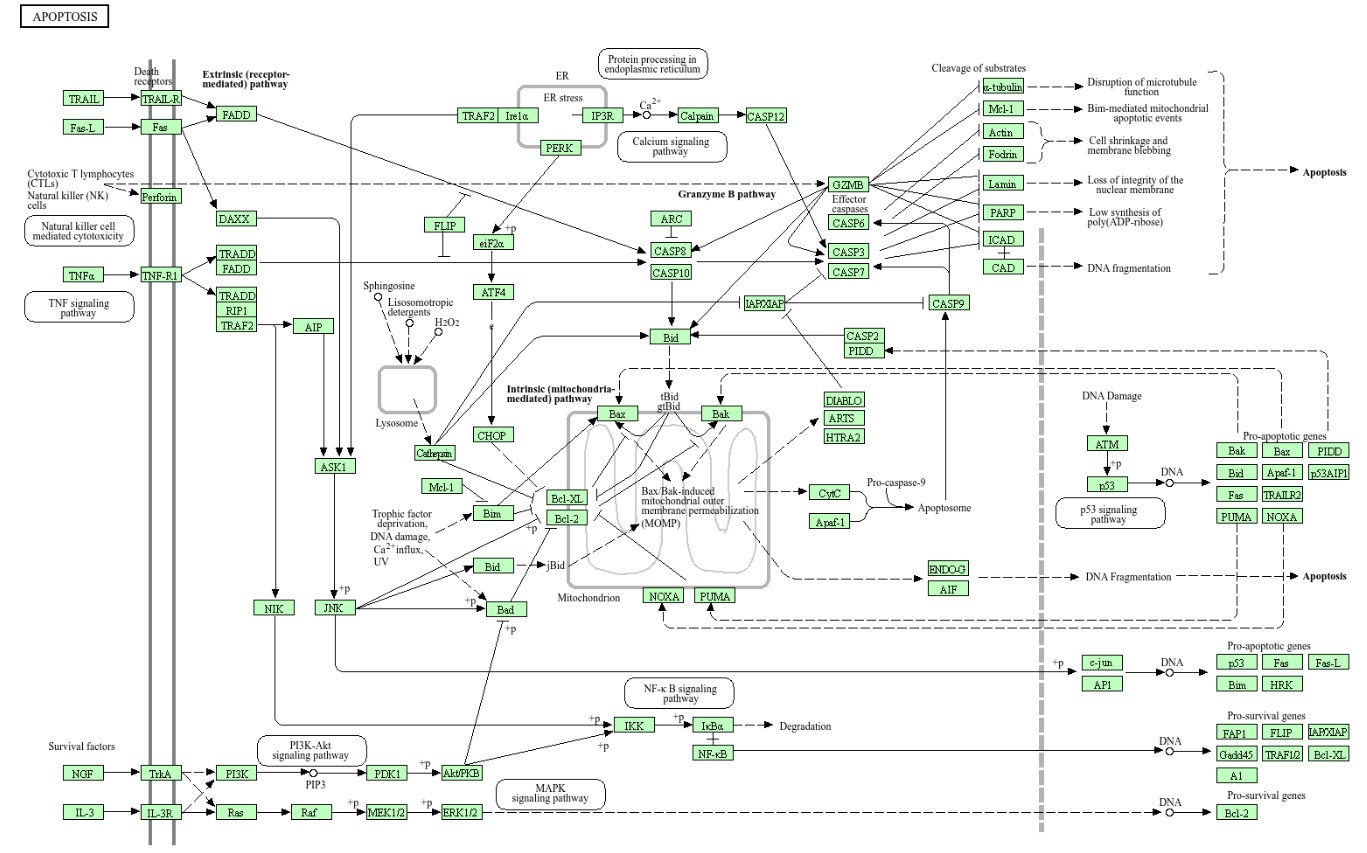


SF 4: KEGG pathway of Apoptotic cell death


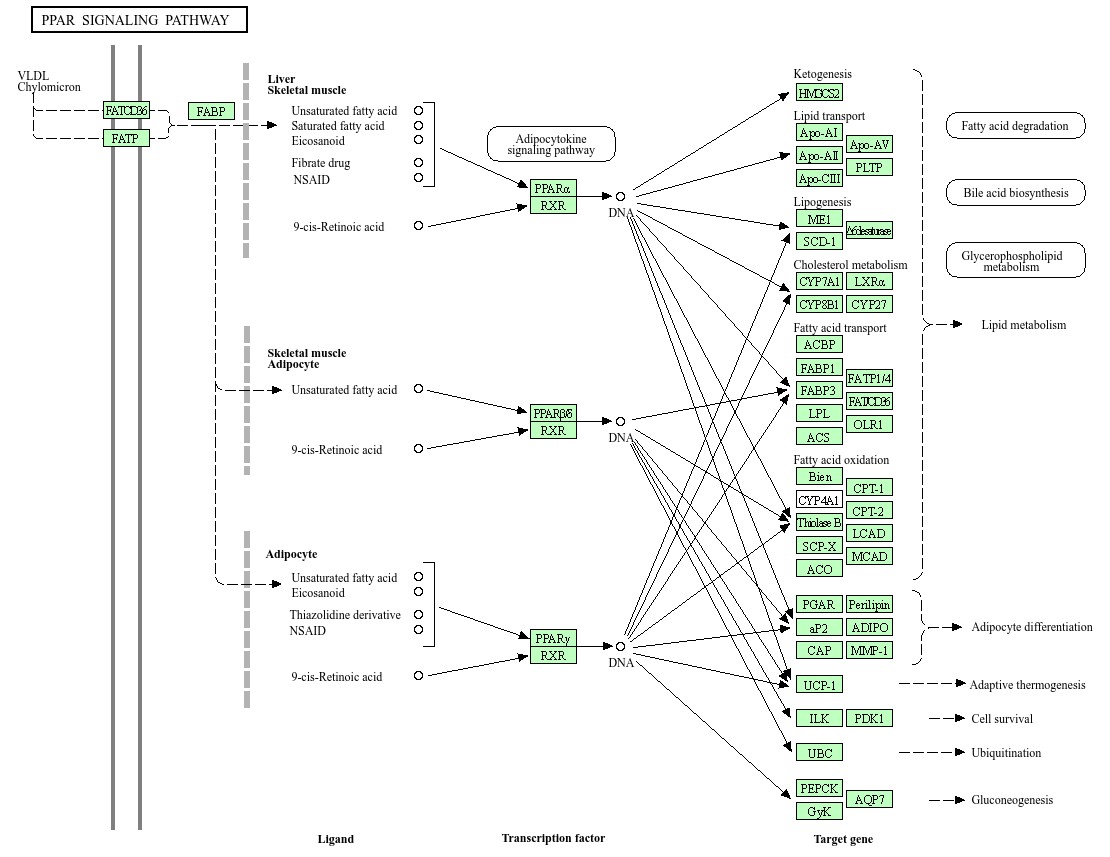


SF 5: KEGG pathway of PPAR signaling


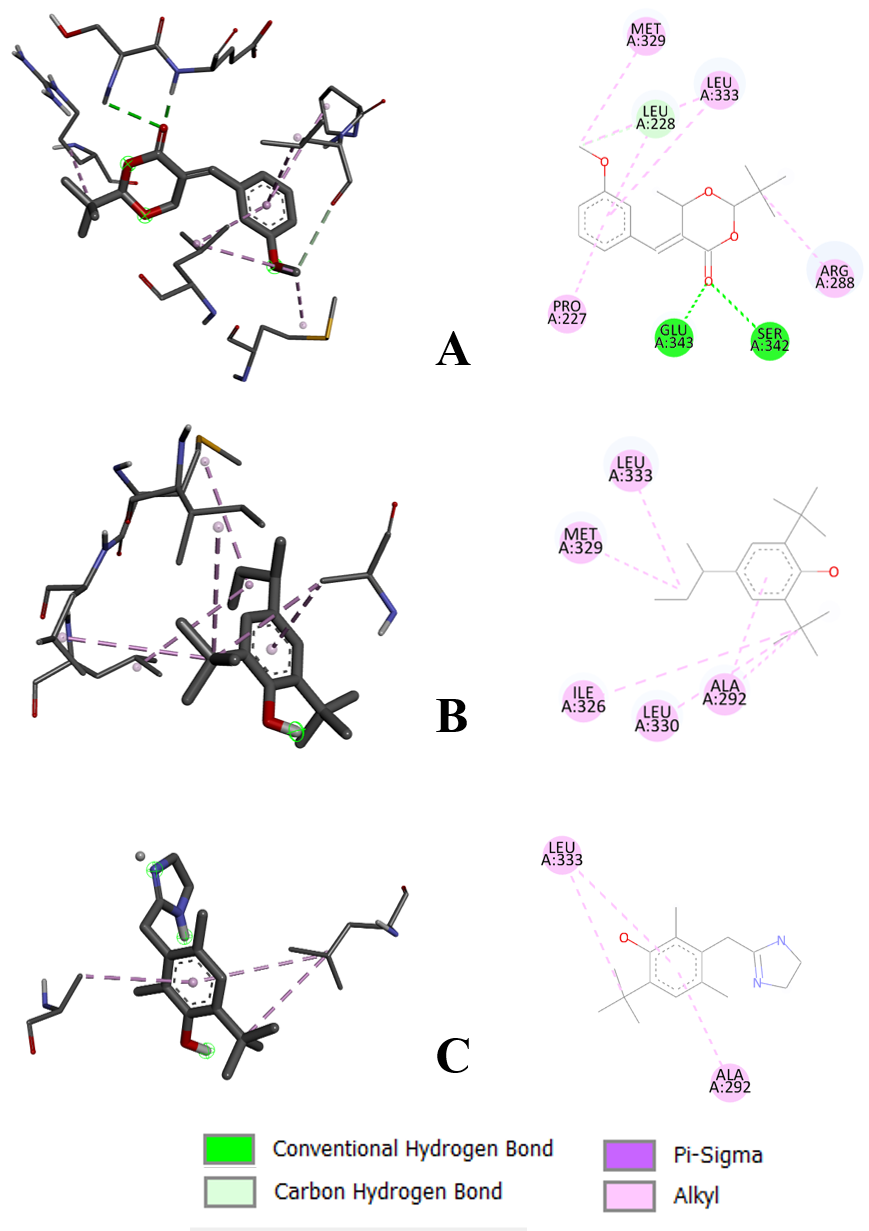


SF 6: 2D and 3D views of compounds' interaction with PPAR-γ. A. 2-tert-butyl-5-[(3-methoxyphenyl) methylidene]-6-methyl-1,3-dioxan-4-one (5373757) B. 4-butan-2-yl-2,6-ditert-butylphenol (86583), C. 6-tert-butyl-3-(4,5-dihydro-1H-imidazol-2-ylmethyl)-2,4-dimethylphenol (4636).


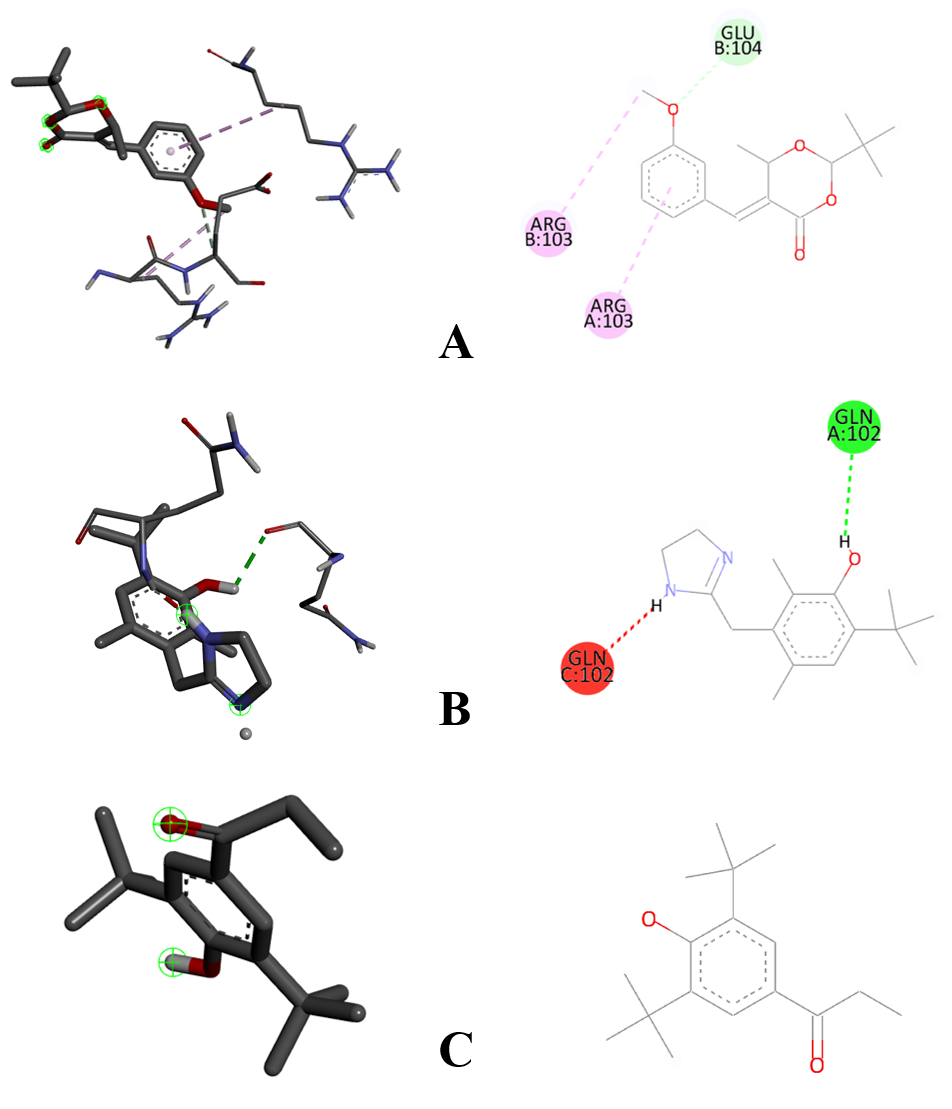


SF 7: 2D and 3D views of compounds' interaction with TNF-α. A. 2-tert-butyl-5-[(3-methoxyphenyl) methylidene]-6-methyl-1,3-dioxan-4-one (5373757) B. 6-tert-butyl-3-(4,5-dihydro-1H-imidazol-2-ylmethyl)-2,4-dimethylphenol (4636), C. 1-(3,5-ditert-butyl-4-hydroxyphenyl) propan-1-one (616172).


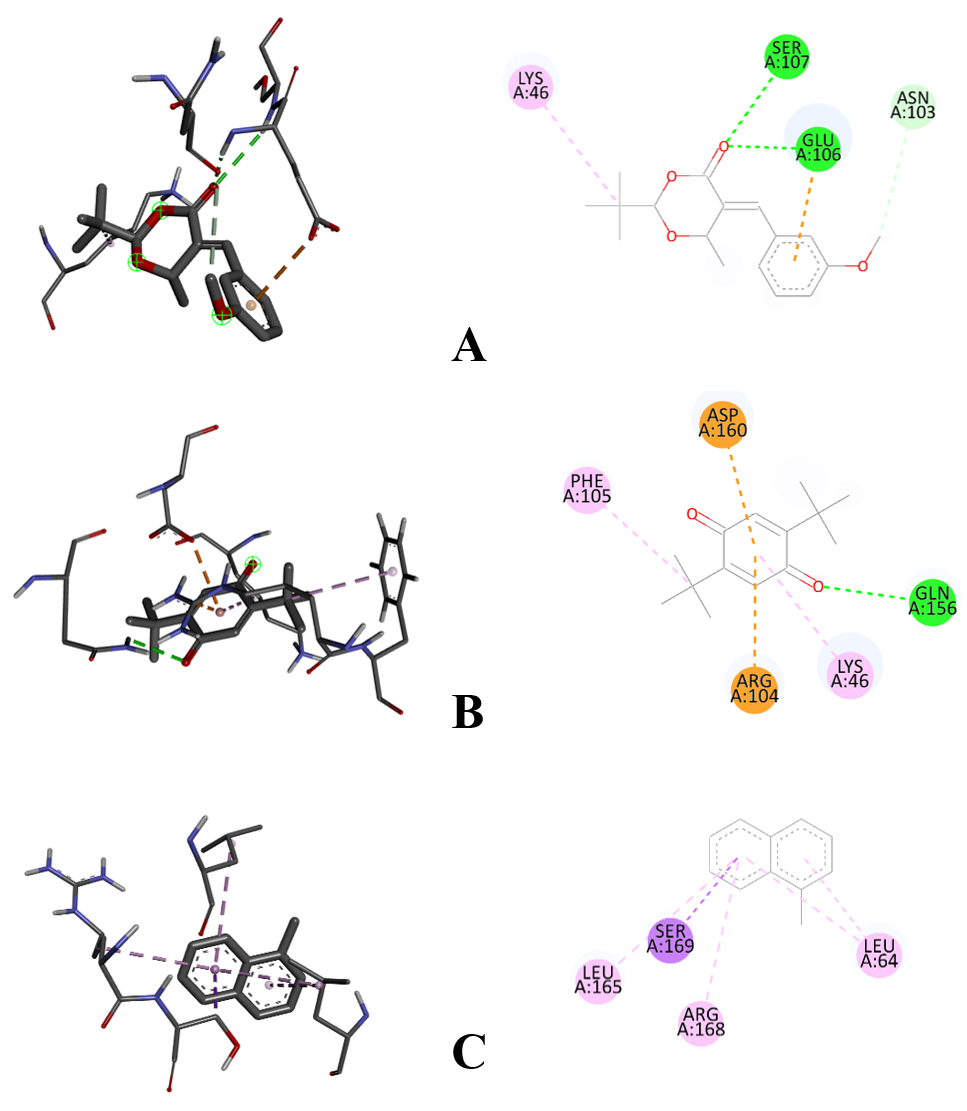


SF 8: 2D and 3D views of compounds' interaction with IL-6. A. 2-tert-butyl-5-[(3-methoxyphenyl) methylidene]-6-methyl-1,3-dioxan-4-one (5373757) B. 2,5-di*tert*-butylcyclohexa-2,5-diene-1,4-dione (17161), C. 1-(3,5-ditert-butyl-4-hydroxyphenyl) propan-1-one (616172) 1-methylnaphthalene (7002).
